# Expression of the SIBLINGs and their MMP partners in human benign and malignant prostate neoplasms

**DOI:** 10.18632/oncotarget.10110

**Published:** 2016-06-16

**Authors:** Charles C. Anunobi, Komal Koli, Geetu Saxena, Adekunbiola A. Banjo, Kalu U.E. Ogbureke

**Affiliations:** ^1^ Department of Anatomic and Molecular Pathology, College of Medicine, University of Lagos, Lagos, Nigeria; ^2^ Department of Diagnostic and Biomedical Sciences, The University of Texas Health Science Center at Houston, School of Dentistry, Houston, Texas, U.S.A

**Keywords:** SIBLINGs, dentin sialophosphoprotein, prostate cancer, matrix metalloproteinases, SIBLING-MMP interaction

## Abstract

The small integrin binding ligands n-linked glycoproteins (SIBLINGs) have emerged as potential diagnostic and prognostic indices, and as key targets, in cancer therapy. Three members of the SIBLING family: bone sialoprotein (BSP); osteopontin (OPN); and dentin matrix protein1 (DMP1), bind and interact with specific matrix metalloproteinases (MMPs): BSP-MMP2; OPN-MMP3; DMP1-MMP9, in biochemical and biologic systems. The other two family members are dentin sialophosphoprotein (DSPP) and matrix extracellular phosphoglycoprotein (MEPE). The specific SIBLING-MMP pairing reported in some cancers have not been reported in prostate neoplasms. In this study, we investigated SIBLING-MMP expression and potential interaction in prostate neoplasms. Chi square analysis of immunohistochemistry results showed significant upregulation of OPN (X^2^=25.710/p<0.001), BSP (X^2^=19.546/p<0.001), and DSPP (X^2^=8.720/p=0.003) in prostate adenocarcinoma (pAdC). MEPE was significantly upregulated in benign prostate hyperplasia (BPH; X^2^=44.153/p<0.001). There were no significant differences in MMP expression between BPH and pAdC. Western blot analysis showed significantly elevated BSP and DSPP in prostate cancer-derived cells. Immunofluorescence studies confirmed BSP-MMP2, OPN-MMP3, and DMP1-MMP9 coexpression in two cancer-derived cell lines, whereas in situ proximity ligation assays confirmed potential BSP-MMP2, OPN-MMP3, and DMP1-MMP9 interactions in BPH and pAdC. Our reports provide evidence that SIBLING-MMP interaction may play a role in the progression of BPH to pAdC.

## INTRODUCTION

Worldwide, prostate cancer remains the most frequently diagnosed cancer and the sixth leading cause of death amongst males [1, 2]. In the United States, prostate cancer diagnosis ranks second only to cutaneous malignancy [3, 4]. Men of African and Afro-Caribbean descents have the highest incidence of, and mortality rate from, prostate cancer [5–7]. It is estimated that about 230,000 men will be diagnosed with prostate cancer out of which approximately 28,000 will die from the disease [1, 3, 8, 9]. Although the advent and use of prostate specific antigen (PSA) as “marker’ and a screening tool of diseases progression has aided the early detection of prostate cancer, its shortfall remains the lack of robustness in discriminating indolent from aggressive disease [4, 10, 11]. A significant proportion of prostate neoplasms grow quite slowly, remaining asymptomatic and indolent for many years, often never progressing to metastatic disease [9, 12]. Thus, the implication that an estimated 1,000 men would have to be screened using the PSA criteria in order to prevent one death from prostate cancer, is increasingly proving to be less cost-effective [13]. Later established indices that complement the PSA test, such as the D'Amicio risk stratification categories and the Gleason score from prostate biopsy, do not possess sufficient accuracy to robustly predict which neoplasia is likely to progress, or continue to remain indolent [9, 12, 14]. The need to identify independent and more accurate predictive markers of enhanced clinical utility in the treatment of prostate cancer patients therefore remains [9].

In recent years, not only have the SIBLING (small integrin binding ligand n-linked glycoprotein) family of extracellular matrix proteins been detected in various human cancers, they also have been characterized as key players in the various stages of cancer progression [15–20]. The SIBLINGs have therefore emerged as proteins with potential diagnostic and prognostic utility, and as new therapeutic targets [20]. The five currently known members of the family are bone sialoprotein (BSP), osteopontin (OPN), dentin matrix protein 1 (DMP1), dentin phosphoglycoprotein (DSPP), and matrix extracellular phosphoglycoprotein (MEPE; 21).

Within the past decade, our collaborators and we have reported that three members of the SIBLING family bind with high affinity and activate specific pro-matrix metalloproteinases (MMPs): MMP2 with BSP; MMP3 with OPN; and MMP9 with DMP1 [22]. Significantly, these specific interactions and their possible biologic relevance, first demonstrated with purified proteins in biochemical systems, were confirmed to obtain in biologic systems as well [22–24]. In some cancers the SIBLING-MMP coexpression correlated with tumor aggressiveness, poor prognosis, or both [25–27].

In the current study, we performed immunohistochemistry (IHC) and in situ proximity ligation assay (iPLA) on archived human tissue sections of benign prostate hyperplasia (BPH) and prostatic adenocarcinoma (pAdC). Western blot, quantitative real time (q RT) PCR, and immunofluorescence analyses were performed on various human prostate cell lines, in order to investigate the expression of the SIBLINGs and their cognate MMPs in benign and malignant prostate neoplasms.

## RESULTS

### SIBLING-MMP expression in BPH and pAdC

**Figure 1:**
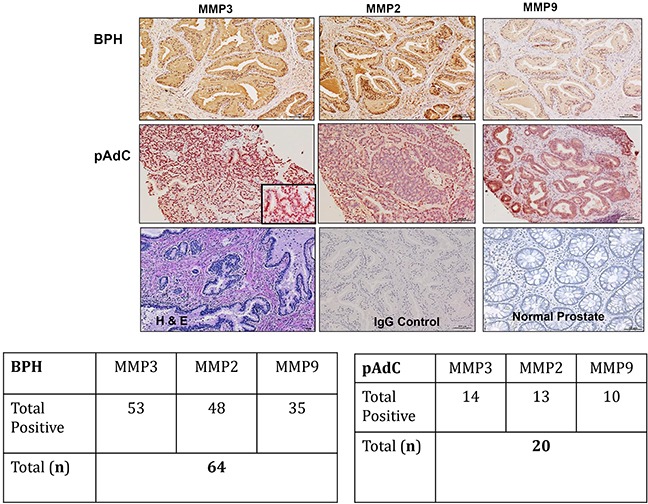
Immunohistochemistry (IHC) on BPH and pAdC tissue sections showing positive immunoreactivity for MMP3, MMP3, and MMP9 Top row panel is representative IHC results (brown/red stain) for the MMPs expression in BPH. Second row represents illustrative positive immunoreactivity (brown/red stain) for the MMPs in pAdC. Third row shows an illustrative H&E section of BPH, experimental negative control consisting of section treated with pre-immune IgG serum, and representative normal prostate tissue showing negative immunoreactivity for all three MMPs. Scale bar, 100μm. Inserted tables summarize the number positive cases of BPH and pAdC for each of the MMPs.

**Figure 2:**
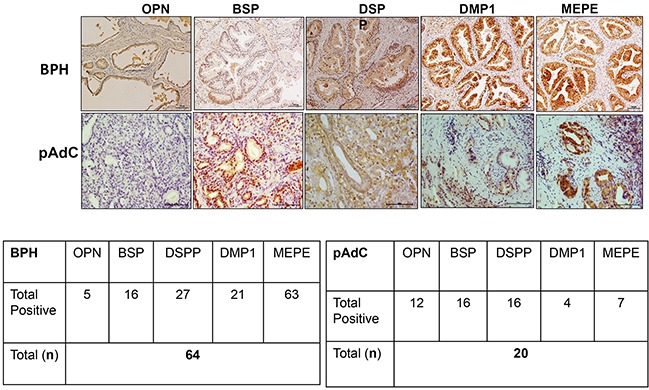
IHC on BPH and pAdC tissue sections showing positive immunoreactivity for the SIBLINGs Top row panel is representative IHC results (brown stain) for SIBLINGs expression in BPH. Second row represents illustrative positive immunoreactivity (bright red/brown stains) for SIBLINGs in pAdC. Experimental and tissue negative controls are as described in Figure 1. Scale bar, 100μm. Inserted tables summarize the number positive cases of BPH and pAdC for each of the SIBLING.

We performed immunohistochemistry on archived pathologic tissue sections of surgically resected BPH and pAdC for SIBLING-MMP expression and surveyed six prostate cell lines for SIBLING-MMP expression by Western blot and quantitative RT-PCR. Representative micrographs of MMP2, MMP3, and MMP9 immunoreactivity of BPH and pAdC are shown in Figure 1, whereas representative examples of SIBLING immunoreactivity are shown in Figure 2. MMP (Figure 1) and SIBLING (Figure 2) immunoreactivity in BPH and pAdC are represented by distinct brown/red staining with cytoplasmic and perinuclear distribution. Punctate nuclear staining was observed in some tumors. Normal prostate gland tissue showed negative immunoreactivity for SIBLINGs and their MMP partners (Figure 1).

As summarized in Figure 1, MMP2 showed positive immunoreactivity in 48 (75%; n=64) cases of BPH and in 13 (65%; n=20) cases of pAdCs. MMP3 immunoreactivity was present in 53 cases (98%) of BPH and 14 cases (70%) of pAdCs, while 35 (54%) cases of BPH and 10 (50%) cases of pAdCs were immunoreactive for MMP9. With respect to the SIBLINGs (Figure 2), the 64 cases of BPH and 20 cases of pAdC surveyed showed immunoreactivity for at least one member of the SIBLING family. OPN was expressed in 5 (8%) of BPHs and 12 (60%) of pAdCs. Positive immunoreactivity for BSP was observed in 10 (16%) BPHs and 16 (80)%) pAdCs. DSPP expression was present in 27(42%) of BPHs and in 16 (80%) pAdCs, while DMP1 expression was recorded in 21 (33%) of BPH and in 4 (20%) of pAdCs. MEPE expression was observed in 63 (98%) of BPH and in 7 (35%) pAdCs, suggesting that MEPE is considerably upregulated in BPHs.

Furthermore, when expression of the cognate MMPs in BPH and pAdC were compared, Chi-square analyses revealed no significant difference for MMP3 (p=0.213), MMP2 (p=0.381) or MMP9 (p=0.714) between BPH and pAdC. On the other hand, significant differences in expression of all but one of the SIBLINGs were noted between BPH and pAdC. There was significant upregulation of OPN (X^2^ = 25.710, p<0.001), BSP (X^2^ = 19.546, p<0.001), and DSPP (X^2^ = 8.720, p=0.003) in pAdC compared with BPHs, whereas MEPE was significantly upregulated in BPH (X^2^ = 44.153, p<0.001) compared with expression in pAdCs. There was no significant difference for DMP1 expression (p=0.274) between BPH and pAdC.

**Figure 3:**
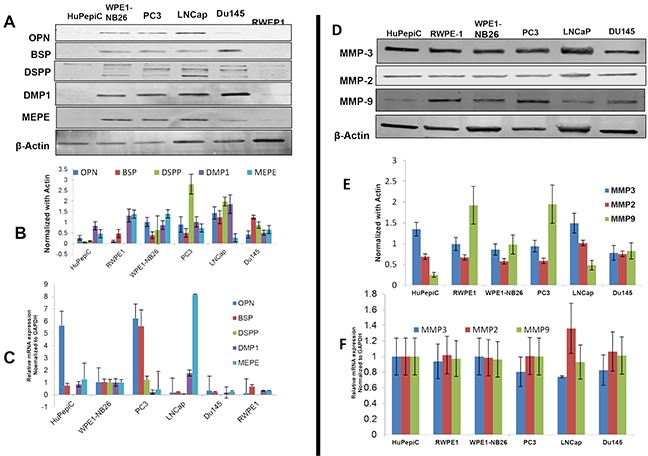
Western blot and quantitative RT-PCR analysis of SIBLING and MMP expression in normal and prostate cancer cell lines **A.** Western blot (WB) and **B.** quantitative (fold change) histogram show significantly higher BSP and DSPP levels in cancer (PC3, LNCap, Du145) than in normal derived prostate cells (HuPepiC, RWPE1, and WPE1-NB26). **C.** mRNAs of BSP, OPN, and MEPE are detectable in normal and cancer derived prostate cells with significantly high levels of OPN mRNA in HuPepiC and PC3 cell, BSP mRNA in PC3, and MEPE mRNA in LNCaP. DMP1 mRNA was undetectable in DU145 cells, and DSPP mRNA was undetectable in normal-derived prostate cell, HuPepiC and RWPE-1, and cancer-derived DU145. **D.** WB and quantitative histogram **E.** show markedly higher levels of MMP9 in PC3 and HuPepiC than in other cells examined. MMP3 levels are much higher in HuPepiC and LNCap than in other cells. There is no significant difference in MMP2 levels in both normal and cancer derived cells. **F.** qRT-PCR histogram shows marked levels of all three MMPs without any significant difference between normal and cancer derived cells. For WB, β-actin was used as the normalization control, and for qRT-PCR, GAPDH was used as the endogenous control. Values are mean ± SE, n=3. Data are representative of three independent experiments.

As shown in Figure 3 western blot analysis, all five SIBLING family members were present in all six cell lines, normal and cancer derived, although markedly lower/basal levels of each was noted in the normal derived (HuPepiC, RWPE1, WPE1-NB26) cells compared to cancer derived (PC3, LNCap, Du145) cells (Figures 3A, 3B). Notably, DSPP levels were significantly higher in cancer derived cells, PC3 and LNCap, than in the rest of the cell lines (Figures 3A, 3B). mRNA levels of OPN were distinctly higher in HupepiC and PC3 cells, while barely detectable in the other four cell lines, whereas BSP level was distinctly high in PC3 cells than in other cell lines (Figure 3C). MEPE mRNA level in LNCap cells was distinctly high while barely detectable, or very low, in other cell lines. Except for the PC3 and WPE1-N26 cells, where DSPP mRNAs were detectable, DSPP mRNAs were undetectable in the other four cell lines (Figure 3C). DMP1 mRNAs were not detectable in all cell lines except for WPE1-NB26 and LNCap where levels were low but detectable (Figure 3C).

With respect to the MMPs, western blot and quantitative RT-PCR analysis showed markedly high but without significant differences in expression levels of each MMP between normal and corresponding cancer derived prostates cells (Figures 3D-3F). A comparatively higher level of MMP9 protein is however noted in RWPE-1 (normal-derived) and PC3 (cancer-derived) cells than in the rest of the cell lines (Figures 3D, 3E).

### Co-expression of SIBLINGs and their cognate MMPs in BPH and pAdC

**Figure 4:**
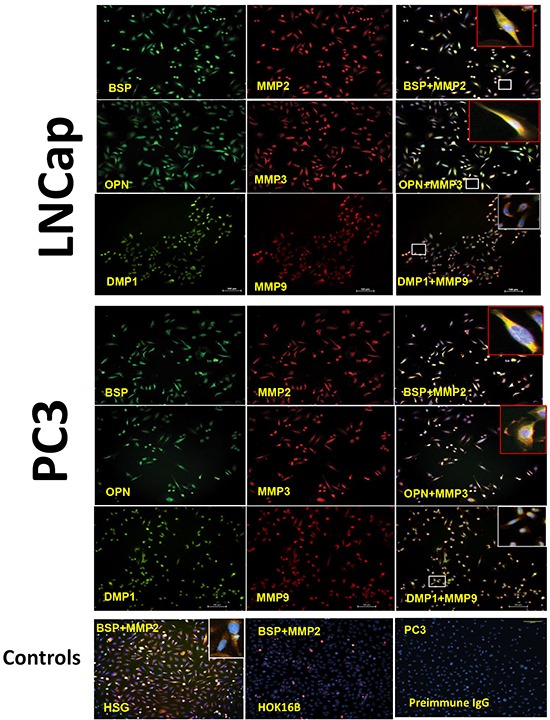
Co-localization of the SIBLINGs and their cognate MMPs in prostate cancer cell lines Distribution of SIBLINGs (green) and MMPs (red) in LNCap and PC3 as determined by immunofluorescence (IF), indicate strong signals in both cancer cell lines. The merged insets evidenced co-localization (yellow) of BSP, DMP1, and OPN with their specific cognate MMP partner (BSP-MMP2, OPN-MMP3, DMP1-MMP9). Signal detected with a fluorescent microscope. Scale bar, 100μm. Controls consisted of normal human salivary gland derived cells (HSG) known to express the SIBLINGs and their cognate MMPs. Experimental negative control consisted of preimmune IgG-treated PC3 cells, whereas tissue-type negative control consisted of HOK 16B cell known to lack SIBLING and MMP expression.

Based on reports of the binding of three SIBLINGs to, and activation of, specific MMPs (BSP/MMP-2, OPN/MMP-3, DMP1/MMP-9) in biochemical and biologic systems [22–24], we hypothesized that the three SIBLINGs with cognate MMP partners (BSP-MMP2, OPN-MMP3, DMP1-MMP9) also colocalize, and potentially interact, in prostate neoplasms. As shown in Figure 4, representative immunofluorescence results show BSP-MMP2, OPN-MMP3, and DMP1-MMP9 coexpression in PC3 and LNCap prostate cancer-derived cell line. Positive controls consisted of normal human salivary glands (HSG) known to coexpress SIBLING-MMP pairs (BSP-MMP2 illustrated lowest row panel, Figure 4), whereas negative cell control consisted of immortalized human oral keratinocyte (HOK16B) cells established to be negative for the SIBLINGs and their cognate MMPs [29]. Experimental negative control consisted of PC3 cells where preimmune IgG antibody substituted for the SIBLINGs and MMPs. (Figure 4).

### Potential SIBLING-MMP interaction in in BPH and pAdC

**Figure 5:**
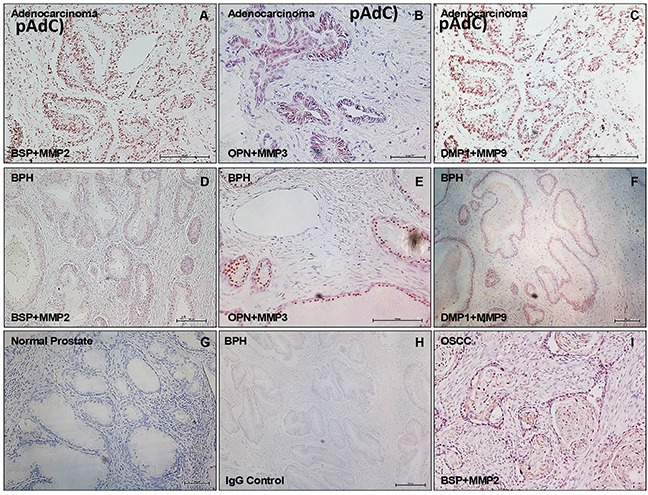
SIBLING-MMP interaction in prostatic neoplasms Top row panel is representative in situ proximity ligation assay (iPLA) showing SIBLING-cognate MMP in pAdC (Figures 5A-5C; red/brown dots), whereas middle row shows SIBLING-cognate MMP in BPH (Figures 5D-5F; red/brown dots). Third row show illustrative tissue negative control (normal prostate; Figure 5G), experimental negative control (BPH treated with pre-immune IgG; Figure 5H), and tissue positive control (human oral squamous cell carcinoma, OSCC; Figure 5I) known to co-express SIBLINGs and their cognate MMPs. Detection was with fluorescent/brightfield microscopy (20X). SIBLING-MMP interaction appears to be more intense in pAdC than in BPH. Other (non-cognate) SIBLING-MMP pairings were negative (see Supplementary Figure). Scale bar, 100 μm. Scale bar, 100μm.

To investigate the potential interaction of SIBLINGs with their specific MMP partners in prostate neoplasms, we performed in situ proximity ligation assay (iPLA) on tissue sections of BPH and pAdC. Figure 5 shows cytoplasmic, perinuclear, and punctate signals in tumor cells, indicating BSP-MMP2, OPN-MMP3, and DMP1-MMP9 colocalization and interaction in pAdC (Figures 5A-5C) and BPH (Figures 5D-5F). Significantly, the SIBLING-MMP interaction pair was specific (BSP-MMP2, OPN-MMP3, DMP1-MMP9), and precluded interaction with non-cognate pairs (Supplementary Figure S1) as previously reported [22, 28]. Tissue positive control consisted of oral squamous cell carcinoma (OSCC) sections known to coexpress cognate SIBLING-MMP partners [28; Figure 5G], while tissue negative control consisted of normal prostate tissue section known to lack tissue expression of the three SIBLINGs with known specific cognate MMP partners (Figure 5H). Experimental negative control consisted of BPH treated with preimmune IgG antibody in place of SIBLINGs and MMPs (Figure 5I).

## DISCUSSION

This is the first study investigating the expression and colocalization of the SIBLINGs and their cognate MMPs in BPH, pAdC, and in normal-derived and cancer-derived prostate cells. Our results indicate that BPHs and pAdCs tissues as well as normal and cancer derived prostate cell lines expressed the SIBLINGs and their three known cognate MMPs. Furthermore, our results confirm the specific SIBLING-MMP interaction first observed in biochemical system using purified proteins [22, 33], and subsequently in biologic systems that include other cancers and highly metabolically active duct epithelia [23, 24, 26]. Although earlier reports have indicated that some members of the SIBLING family are upregulated in prostate cancers, to our knowledge, this is the first report of the co-expression and potential interaction of the SIBLINGs and their specific cognate MMPs in prostatic neoplasms and cell lines.

Others and we have reported the up-regulation of some members of the SIBLINGs in different tumors [15, 26, 30, 31]. Reports also indicate the up-regulation of MMP2, MMP3, and/or MMP9 to be associated with tumor aggressiveness [25–27, 32], suggesting that SIBLING-MMP complexes may facilitate tumor cell metastasis [20]. In addition to the MMPs, SIBLINGs interact with multiple binding partners that include proteases and cell surface receptors in the course of fulfilling an active role in tumor progression [20].

As further substantiated by our present data, different cancer types exhibit different patterns of individual SIBLING expression in tissue sections [4], and serum assays of different cancers, including prostate cancers, indicate similarly [4, 33, 34]. Although the complete lack, or basal level expression, of SIBLING in normal prostate tissue compared to the basal-to-modest levels (protein and mRNA) in normal prostate derived cells (HuPepiC, RWPE1, WPE1-NB26) observed in our study appears inconsistent, this may be attributable to biologic processes of immortalization involved in establishing cell lines.

Nevertheless, the upregulation of the SIBLINGs and their MMP binding partners in several of these cancers consistently tend to portend late stage disease, tumor aggressiveness, and poor prognosis [4, 15, 26, 27]. Our current results from immunohistochemistry analyses showing significant upregulation of BSP, and DSPP in pAdC compared with BPH tissues strongly suggest their potential utility as diagnostic and prognostic markers of disease progression. The distinctly significant upregulation of MEPE in BPHs casts MEPE as a potentially specific diagnostic marker for BPH. On the other hand, the lack of statistically significant differences in the levels of the SIBLING partnering MMPs between BPH and pAdC suggests that levels of these MMPs alone may not be of potential diagnostic and prognostic utility in prostate neoplasms.

Combined, our immunofluorescence and iPLA results provide strong evidence of coexpression, colocalization, and interaction of the three SIBLINGs with known specific MMP partners: BSP-MMP2; OPN-MMP3; and DMP1-MMP9, in prostate neoplasms. Our recent report detailed the iPLA method for the detection of the SIBLING-MMP complex in oral cancer tissues and cell lines [28]. As a powerful technique for detecting multiple proteins sufficiently proximate as to permit protein–protein interactions, the iPLA results in our current study support the presence of specific SIBLING-MMP interacting complexes previously observed in biochemical system, other cancers, and other biologic systems [22–24, 26] to be present in prostate neoplasms. Beyond the scope of our present study, on-going studies will attempt to decipher the mechanisms, significance, and functional role of the SIBLING-MMP interaction in prostate neoplasms.

Thus, it is foreseeable that the expression of the SIBLINGs and their cognate MMP in prostate neoplasms may serve to segregate indolent from progressing disease. Furthermore, combined with serum levels and other indices such as PSA levels as previously reported [4], SIBLING-MMP pairs eventually may emerge as a powerful and more reliable model index for diagnosis, disease progression, and outcome.

In summary, our report provides not only evidence of SIBLING-MMP co-localization in benign and malignant prostate neoplasm, but demonstrates, for the first time, evidence of potential SIBLING-MMP interaction in prostate neoplasm. Given, particularly, the consistent upregulation of BSP and DSPP in prostate cancer, it is plausible to suggest that BSP and DSPP levels may predict progression of benign prostatic neoplasms to malignant disease. Future longitudinal prospective cohort studies of BSP and DSPP expression in a large patient population with BPH and pAdC will be required to determine their utility in the diagnosis of prostatic neoplasms, as well as their utility as therapeutic target and outcome determinant in patients with prostatic neoplasms. It is also anticipated that the results of such studies will uncover any correlation of BSP and DSPP expression with prevailing diagnostic and prognostic indices such PSA and Gleason score, and highlight the complementary strength of BSP and/DSPP to these prevailing indices.

## MATERIALS AND METHODS

### Cell Lines, culture conditions, and antibodies

All cell lines used in the present study were purchased from American Type Culture Collection (ATCC, Manassas, VA, USA). The prostate cancer and other cells lines as well as the antibodies used in this study have previously been published (summarized in Table 1), and are available commercially. Human normal primary prostate epithelial cells, HuPepiC, were maintained in Prostate epithelial cell basal medium (cat # PCS-440-030) supplemented with Prostate epithelial cell growth kit (PCS-440-040) and 1% penicillin/ streptomycin (cat # 15140-122, Life Technologies, Grand Island, NY, USA). RWPE-1 (cat # CRL-11609) cells were derived from histologically normal peripheral human prostate and transfected with HPV-18. WPE1-NB26 (cat # CRL-2852) cells were derived from RWPE-1 cells by N-methyl-N-nitrourea (MNU). RWPE-1 and WPE1-NB26 were maintained in Keratinocyte serum free medium (cat # 17005-042, Life Technologies, Grand Island, NY, USA) containing Bovine Pituitary extract and human recombinant Epidermal Growth Factor supplemented with 1% penicillin/ streptomycin. Prostate adenocarcinoma cell line, PC3 (cat # CRL-1435) was derived from bone metastasis and maintained in DMEM/F-12 medium (ATCC 30-2004) supplemented with 10% FBS (cat # F2442, Sigma-Aldrich, S. Louis, MO, USA) and 1% penicillin/ streptomycin. LNCaP and DU145 are prostate carcinoma cell lines derived from metastatic sites: left supra-clavicular lymph node and brain, respectively. LNCap cells were maintained in RPMI-1640 (ATCC 30-2001) supplemented with 10% FBS and 1% penicillin/ streptomycin, whereas DU145 cells were maintained in Eagle's minimum essential medium, EMEM (ATCC 30-2003) supplemented with 10% FBS and 1% penicillin/ streptomycin.

**Table 1:** Antibodies used in this study

Antibody	Catalog #	Concentration	Isotype	Source
**OPN** **(LFMb-14)**	sc-73631	IHC 1:100 WB 1:2500	Mouse IgG_2b_	Santa Cruz Biotechnology, Dallas, TX
**BSP** **(LFMb-25)**	sc-73630	IHC 1:100 WB 1:1000	Mouse IgG_1_	Santa Cruz Biotechnology, Dallas, TX
**DSPP** **(LFMb-21)**	sc-73632	IHC 1:100 WB 1:500	Mouse IgG_2b_	Santa Cruz Biotechnology, Dallas, TX
**DMP1** **(LFMb-31)**	sc-73633	IHC 1:100 WB 1:1000	Mouse IgG_1_	Santa Cruz Biotechnology, Dallas, TX
**MEPE** **(LFMb-33)**	sc-73635	WB 1:1000	Mouse IgG_1_	Santa Cruz Biotechnology, Dallas, TX
**MEPE** **(LF-155)**		IHC 1:100	Rabbit polyclonal	
**MMP-2**	sc-8835R	WB 1:1500	Rabbit polyclonal	Santa Cruz Biotechnology, Dallas, TX
**MMP-2** **(LF-183)**		IHC 1:100	Rabbit polyclonal	
**MMP-3**	sc-6839R	WB 1:1500	Rabbit polyclonal	Santa Cruz Biotechnology, Dallas, TX
**MMP-3** **(LF-182)**		IHC 1:100	Rabbit polyclonal	
**MMP-9**	sc-21733	WB 1:1500	Mouse IgG_1_	Santa Cruz Biotechnology, Dallas, TX
**MMP-9** **(LF-184)**		IHC 1:100	Rabbit polyclonal	
**Beta-actin**	ab3280	WB 1:5000	Mouse IgG_1_	Abcam, Cambridge, MA
**IRDye anti-Rabbit**	926-32213	WB 1:15000	IgG (H + L)	Li-Cor, Lincoln, NE
**IRDye anti-Mouse**	926-68022	WB 1:15000	IgG (H + L)	Li-Cor, Lincoln, NE

### Case selection for immunohistochemistry

Sixty four (n=64) cases of completely de-identified human BPH and 20 (n=20) pAdCs were selected from archived pathologic tissues of patients retained at the Department of Morbid Anatomy, Lagos University Teaching Hospital (LUTH), Lagos, Nigeria, following appropriate institutional board approval. A retrospective cohort analysis of the immunoreactivity of all five SIBLINGs (BSP, DMP1, DSPP, OPN, MEPE) and three known cognate MMPs (MMP2, MMP3, MMP9) partners were carried out on surgical biopsy specimens of selected cases of human BPH and 20 pAdC. Inclusion criteria for selection were de-identified sample and the presence of histologically diagnosed BPH or pAdC on hematoxylin and eosin (H&E)–stained sections. Diagnosis of either BPH or pAdC on H&E stained microslides was reconfirmed by one of the authors (CA), who also is a consultant pathologist (LUTH), using established architectural and cytologic criteria.

### Immunohistochemistry

Employing the Intellipath FLX automated system (Biocare Medical, Concord, CA, USA) in conjunction with the Intellipath FLX universal HRP-detection kit (IPK5011, Biocare Medical), standard immunoperoxidase techniques were performed on formalin fixed, paraffin-embedded BPH and pAdC sections (~5μm) as recently described [29]. Briefly, sections were deparaffinized in xylene and rehydrated through series of ethanol and water before carrying out antigen retrieval. Endogenous peroxidase quenching was carried out for 10min. To reduce nonspecific binding, sections were treated with background punisher (catalog # BP974H; Biocare Medical) for 20min. Thereafter, sections were loaded onto the preprogrammed and timed autostainer for sequential primary and secondary antibody incubation. Chromogen detection was performed with either 3,3′-diaminobenzidine (BDB2004), or Warp Red (WR806H). Sections were counterstained with hematoxylin for 10sec. Negative control sections were treated with Universal Negative Control Serum (NC498, Biocare Medical). Representative photographic images were captured using Eclipse Ni-E microscope with Nikon DS-U3 digital camera and NIS Elements AR software (Nikon, Melville, NY, USA).

### Scoring of immunohistochemistry results

Semiquantitative scoring methods for immunostaining have been described [23, 27]. Briefly, following established and calibrated criteria, semiquantitative scores of SIBLING/MMP immunoreactivity was assigned as follows by two of the authors (KK and KUO): 0 (negative; not detectable or faint staining, <10% of tumor cells); 1 (10% to 50% immunoreactive tumor cells); 2 (50% to 75% immunoreactive tumor cells); and 3 (widely/intensely expressed in tumor cells). Interoberver differences were reconciled through microscopic conferencing.

### Western blotting

Western blot (WB) was performed on whole cell lysates of prostate cancer and appropriate control cell lines to determine protein expressions with 4-20% Criterion TGX (567-1094, Bio-Rad, Hercules, CA, USA) using sodium dodecyl sulfate polyacrylamide gel electrophoresis (SDS-PAGE). Trans-Blot Turbo transfer system (170-4155EDU, Bio-Rad, Hercules, CA, USA) was used to transfer proteins onto low-fluorescence polyvinylidene difluoride (PVDF) membrane (cat# 20130403, Bio-Rad). 5% non-fat milk was used for blocking. Membranes were treated with primary overnight at 4°C, and thereafter with corresponding secondary antibodies for 1h at room temperature. LiCor Odyssey Scanner was used to photograph the blots and actin was used to normalize the intensities.

### Real-Time PCR

Total RNA was extracted from cell lysates of prostate cancer and appropriate controls using Trizol reagent (cat # 15596-026, Life technologies). Nano drop machine was used to determine concentration of RNA in samples. All primers were purchased from IDT (Integrated DNA technologies) and are listed in Table 2. iScript RT supermix (cat# 1708841, Bio-Rad) was used to reverse transcribed total RNA. Quantitative real-time PCR was performed using iTaq UniverSYBR Green PCR Master mix (cat# 1725124, Bio-Rad). mRNA levels were normalized to GAPDH and analyzed using Bio-Rad CFX manager software.

**Table 2:** Primers used for RT-PCR

		List of Primers
**hOPN**	Forward Reverse	CAAACGCCGACCAAGGAAAAC TGGCCACAGCATCTGGGTAT
**hBSP**	Forward Reverse	TTCCAGTTCAGGGCAGTAGT AGCCCAGTGTTGTAGCAGAAA
**hDSPP**	Forward Reverse	AAAAGTCCAGGACAGTGGGC GCTTTGAGGAACTGGAATGGC
**hDMP1**	Forward Reverse	CCTGTGCTCTCCCAGTAACC ATTTGCCAAGGGTGGTGTTG
**hMEPE**	Forward Reverse	CCGGCAGCTATCCACACCAG GAAATGTTGGTGCTGCCCAGG
**hMMP2**	Forward Reverse	GGAGCTCTATGGGGCCTCTC GTCACAGTCCGCCAAATGAAC
**hMMP3**	Forward Reverse	TTTAAAGGAAATCAATTCTGGGCT CCTGGCTCCATGGAATTTCTC
**hMMP9**	Forward Reverse	CGCCTCTGGAGGTTCGACG GAAGCGGTCCTGGCAGAAAT
**hGAPDH**	Forward Reverse	CTCCTCCGGGTGATGCTTTT ACATGTAAACCATGTAGTTGAGGTC

### Immunofluorescence

Prostate cancer and control cell lines plated on coverslips in a 6-well dish overnight were washed 3x with 1XPBS and fixed in 3% paraformaldehyde for 20min before permeabilization in 0.1% tritonX-100 for 10min. Cells were treated with blocking buffer (1XPBS, 3% goat serum) for 1h at room temperature, followed by overnight incubation at 4°C with primary antibodies to SIBLING/MMP diluted in blocking buffer. Coverslips were washed 3x with 1XPBS and incubated with secondary antibodies for 1h before mounting with Prolong Gold antifade reagent with DAPI (catalog no. P36931, Life Technologies).

### In situ proximity ligation assay (iPLA)

iPLA was used to verify cellular interactions of the SIBLINGs with known cognate MMP partners (BSP-MMP2, OPN-MMP3, and DMP1-MMP9) in human prostate carcinoma tissue sections. Prior to iPLA, BPH/pAdC tissue sections were deparaffinized and antigen retrieved. iPLA was performed using the HRP/NovaRed detection kit from Olink Bioscience according to anufacturer's protocol (catalog no. DUO92012, Sigma-Aldrich). Briefly, tissues were incubated with primary anti-MMP polyclonal (rabbit) antibody and anti-SIBLING (mouse monoclonal antibody) or with normal rabbit IgG. They were then incubated with corresponding secondary antibodies conjugated to oligonucleotides PLA probes (MINUS and PLUS) for 1h at 37°C. Rolling circle amplification was performed using T4-ligase (Olink Bioscience, St. Louis, MO, USA). Fluorescent-labeled oligonucleotides (catalog no. DUO92008) and HRP/NovaRed (catalog no. DUO92012; Sigma-Aldrich) were used to detect rolling circle amplification products in tissues, respectively. Protein interactions were observed as red/brown punctate signals and were captured using Nikon Eclipse Ni-E microscope and NIS Elements AR software.

### Statistical analysis

All statistical analyses were carried out using WinPepi 11.5. To determine if there were significant differences in SIBLING and cognate MMP expression between BPH and pAdC, chi square analyses (Fisher's Exact test where indicated) with post-hoc Bonferonni comparisons were used. Other results were analyzed using either Student's t test or One-Way analysis of variance (ANOVA) with subsequent post hoc Tukey's pairwise analysis. Differences were considered significance if p<0.05.

## SUPPLEMENTARY FIGURE


