# Supplementary material for: Expression of the SIBLINGs and their MMP partners in human benign and malignant prostate neoplasms
Source: Oncotarget. 2016 Jun 16;7(30):48038–49. doi: 10.18632/oncotarget.10110 (PMC5216998; doi:10.18632/oncotarget.10110)
Supplement: Supplementary file 1 [file oncotarget-07-48038-s001.pdf]

Expression of the SIBLINGs and their MMP partners in human benign and malignant prostate neoplasms

SUPPLEMENTARY FIGURE

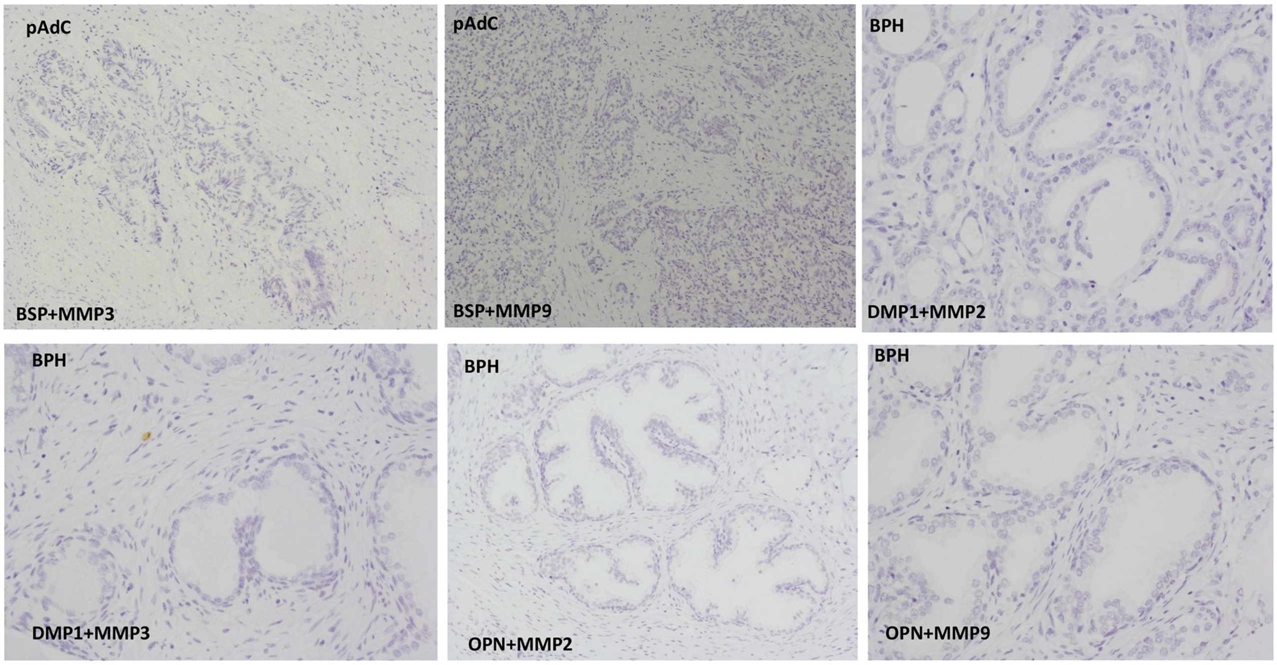

Supplementary Figure S1: iPLA shows negativity in mismatch SIBLING-MMP pairs in prostate neoplasms, compared to result in Figure 5, further validating the specificity of the SIBLING-cognate MMP interaction previously reported in other biologic systems. (Mag. 10X)
